# Opinions of the UK general public and stroke survivors in using artificial intelligence and ‘opt-out’ models of consent in medical research: a qualitative study

**DOI:** 10.1136/bmjopen-2025-108671

**Published:** 2025-12-15

**Authors:** William Heseltine-Carp, Mark Thurtson, Michael Allen, Daniel Browning, Megan Courtman, Aishwarya Kasabe, Emmanuel Ifeachor, Stephen Mullin

**Affiliations:** 1 University of Plymouth, Plymouth, UK; 2 Medical School, University of Exeter, Exeter, UK

**Keywords:** Stroke, Machine Learning, Artificial Intelligence, Stroke medicine

## Abstract

**Abstract:**

**Background:**

Artificial intelligence (AI) in healthcare often requires large, confidential clinical datasets. However, a recent UK government survey revealed that 20–40% of the public remain sceptical of its use in health research due to concerns about data security, patient–practitioner communication and commercialisation of data. A greater understanding of public attitudes is therefore needed, particularly in the context of stroke research.

In this article, we describe the patient and public involvement work undertaken for the AI-Based-Stroke-Risk-fActor-Classification-and-Treatment (ABSTRACT) project, which aims to train AI models to predict future stroke risk from the electronic health records of 1 18 736 patients.

**Aims:**

We aimed to evaluate the opinions of stroke/transient ischaemic attack (TIA) patients, caregivers and members of the public on the following themes: (1) the acceptability of using AI to predict stroke from electronic health records, (2) obtaining these data using an opt-out model of consent and (3) allowing access to this dataset from members both within and outside of the routine clinical care team.

**Methods:**

A total of 83 participants were recruited via the National Health Service social media and by approaching hospital inpatients. Participants were first provided with background information on stroke, AI in medical research and ABSTRACT’s proposed data handling protocol. A mixed methods approach was then used to explore each of the above themes using online survey, semistructured focus groups and one-to-one interviews.

**Results:**

Nearly all participants felt that it was appropriate to use patient data to train AI models to predict stroke risk and that it was acceptable to obtain these data via an opt-out model of consent. Almost all participants also agreed that data could be shared within and outside of the routine clinical care team, provided it was General Data Protection Regulation compliant and used for medical research only.

**Conclusion:**

The public and those with lived stroke/TIA experience appeared to support using deidentified medical datasets for AI-driven stroke risk prediction under an opt-out consent model. However, this is provided that the research conducted is transparent, for a clear medical purpose and adheres to strict data security measures.

**STRENGTHS AND LIMITATIONS OF THIS STUDY**

- Opinions were evaluated in a mixed methods approach by using one-to-one inpatient interviews, focus groups and an online survey, in order to improve the richness of our data and mitigate detection bias.
- The study describes how patient and public involvement can be successfully integrated into projects to produce ethical and General Data Protection Regulation compliant research, fit for confidential advisory group approval. We hope this will be a useful example to readers seeking similar approval.
- The majority of respondents were those with lived experience of transient ischaemic attack/stroke, hence, the views of the wider general public may be under-represented.
- Opinions were evaluated in the context of the AI-Based-Stroke-Risk-fActor-Classification-and-Treatment study; hence, attitudes may not be generalisable to other research areas.

## Introduction

 Patient and public involvement (PPI) describes the process of actively engaging patients, caregivers and the general public in designing, conducting and disseminating research. There are a number of benefits of PPI. First, it provides an opportunity to tailor research to the needs and values of patients. Second, it encourages research to be conducted in an ethical and transparent manner. Third, it enhances trust from patients and the public in research by empowering them to take ownership and an active role in research. Finally, it enables better dissemination and accessibility of results.

Increasingly, evidence of PPI input is often required by funders and ethical review boards.^1^ This further highlights the need for researchers to undertake such work in order to produce ethical and high-quality research.^2^

As healthcare records and investigations become increasingly digitalised, opportunities emerge to analyse these data to improve clinical care delivery and answer research questions. AI techniques such as machine learning (ML) have the capacity to analyse high-dimensional data with thousands of features, so are often better suited to the analysis of large, complex, multifaceted datasets than traditional statistical techniques.

It is likely that artificial intelligence (AI) will continue to accelerate medical research and transform clinical practice.^3 4^ However, with the significant capabilities that AI offers, comes the need for appropriate stewardship and responsibility. Approximately 20–40% of individuals remain sceptical of the use of AI models in medical research.^5^ Due to its complexity, large datasets are often required to train AI models. Since it would be impractical to gain consent from each individual, data are often deidentified, and individuals are excluded on an opt-out basis. Hence, much of this scepticism relates to data acquisition, storage and security of data. Naturally, societal impacts of AI have also been raised, including job security, effective communication with patients and the sale of data/models to external organisations.^6^

The use of routine healthcare data in medical research presents new challenges as well as major opportunities. In order to ensure that the data used is representative, it must be as comprehensively collated as possible. Additionally, in order to undertake such research in an ethical and compliant manner, it is essential to demonstrate a valid medical purpose and confirm support for the use of data without consent among key stakeholders, particularly those with lived experience of the disease and (where the use of ‘control’ data is required) the public at large. High-quality PPI work is therefore an increasingly necessary prerequisite to this type of research.

### Aims

The ^7^AI-Based-Stroke-Risk-fActor-Classification-and-Treatment (ABSTRACT) study aims to train ML models on routine CT head, MRI head, ECG, echocardiogram, laboratory test and medical history data, to predict future stroke risk at 1, 3, 5 and 10 years. Models will be trained using general practice (GP) and hospital records from 9155 stroke cases and 109 581 controls obtained from National Health Service (NHS) sites across Devon and Cornwall in the UK.

In this article, we report the findings of our PPI work for this study, in which we aimed to evaluate the opinion of patients and the public on the following three questions:

1. Is it acceptable to train AI to predict the future risk of stroke from routine hospital data?
2. Is it acceptable to acquire and handle large patient datasets using an ‘opt-out’ model of consent?
3. Is it acceptable for members from (a) within the routine clinical care team, and (b) from outside the routine clinical care team to have access to these datasets?

**Figure 1:**
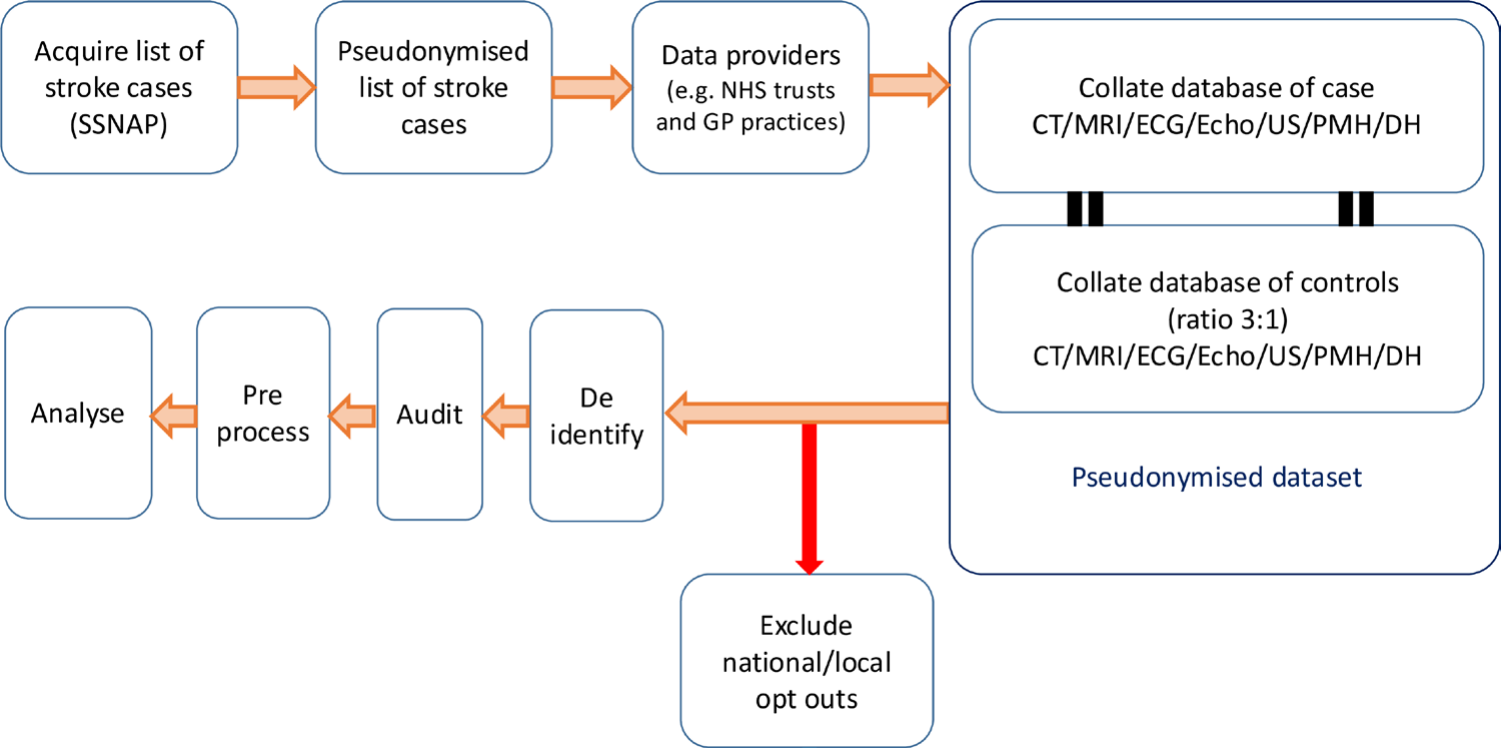
Data acquisition. The figure illustrates the data acquisition and handling process. A detailed description of the process is outlined in the above paragraph above. GP, general practice; NHS, National Health Service; SSNAP, Sentinel Stroke National Audit Programme; US, Ultrasound; PMH, past medical history; DH, drug history.

The details of the ABSTRACT’s protocol and data handling procedures are published elsewhere.^8^ Briefly, figure 1 outlines the data acquisition and handling process. To begin with, cases were identified from the Sentinel Stroke National Audit Programme database,^9^ a mandated national audit of stroke cases in the UK, which contains data related to every inpatient UK stroke admission. This was undertaken by the data controller, a member of the routine clinical care team (ie, an individual who would normally have access to this data for clinical purposes). Based on these data, a pseudonymised list of NHS numbers (the unique identifier assigned to every UK citizen) of stroke cases was generated. This pseudonymised list was then shared with data providers, such as NHS hospitals, integrated care boards and GPs, who then returned relevant data for these cases and controls. The data controller then linked these data to form a single, pseudonymised dataset of cases and controls. Participants were then compared against the national data opt-out database (NDOO)^10^ and removed accordingly. Finally, the dataset was then deidentified by the data controller and shared with the research team for analysis. At all stages, the minimum number of data handlers was given access to the dataset, and data were stored on encrypted NHS computer systems. All data was handled in compliance with General Data Protection Regulation (GDPR) and the University of Plymouth’s ‘research ethics and integrity policy’^11^ and ‘research data management policy’.^12^

## Methods

To ensure a representative sample, we used a mixed methods approach to triangulate data collection. These were an in-person/online (hybrid) semistructured focus group, an online questionnaire and one-to-one interviewing of inpatients at Derriford Hospital, UK. Data was collected between 1 January 2024 and 1 May 2025 and iteratively reviewed until the point of data saturation was reached.

Prior to any of these three approaches, participants were provided with two documents. The first provided background information on the definition of stroke and stroke risk factors, the definition of AI and its roles in stroke research, the aims of the ABSTRACT project, and finally, the purpose of PPI in research (online supplemental file appendix 1). The second provided details on how our study planned to acquire, handle, process and store clinical data (online supplemental file appendix 2). Focus group and one-to-one sessions were facilitated by medical doctors with clinical experience in stroke (but not involved in the participants’ care). At the end of sessions, participants were invited to join a study oversight group.

### Focus group

Those with lived experiences of stroke/transient ischaemic attack (TIA) and members of the public were invited to join two focus groups discussing their opinions on training AI to predict future risk of stroke using routine hospital data. Participants were invited to attend online or in person. This was advertised via social media pages of the University Hospitals Plymouth NHS Trust, Stroke Association^13^ and National Institute for Health and Social Care Research South West Peninsula Clinical Research Network.^14^

Participants were offered a £20 Amazon voucher^15^ for their time and reimbursed for travel expenses. Registration took place via an online survey. Participants were then contacted via email to share the above documentation and session details.

Each focus group consisted of one 90-minute session and used a semistructured discussion guide approach. 10 min were dedicated to a presentation briefly defining and explaining stroke and the use of AI in research. A further 10 min were dedicated to explaining how our study planned to collect, store and analyse data, in addition to explaining how to opt out of the study. Participants were then asked to discuss three questions in groups of three to four. These were: ‘(1) *is it acceptable to collect, store and analyse data in this manner for medical research from: (a) within the routine clinical care team and (b) from outside the routine clinical care team?’;* ‘(2) *has sufficient opportunity for participants to ‘opt out’ of research been given?*’ and (3) *is there anything further you would like to add?’*. For questions one and two, a consensus was reached and summarised as ‘*(YES/NO/UNABLE TO REACH CONSENSUS)*’. Those who responded ‘yes’ to question three were given the opportunity to freely record additional comments or discussion points on the project.

### One-to-one interview

Stroke inpatients and family members were invited for a 10–15 min one-to-one interview on assessing their opinion of using AI to predict future risk of stroke using routine hospital data. Prior to each interview, participants were supplied with the aforementioned documentation, which was further outlined on the day of the interview. Participants were then asked the same three questions as above, summarised with a *‘YES/NO’* answer for questions one and two, along with any additional comments they had in response to question three.

### Online survey

An online survey was designed using ‘Qualtrics XM’.^16^ Data were obtained from the dates from 9 February 2024 to 28 July 2024 from 61 participants. Advertisement was done via the study website and social media posts from the local NHS trust. The survey brief included the same information outlined by the aforementioned documentation. Participants were asked four YES/NO questions and had the option to add further comments to each answer. These consisted of: *‘do you believe these study aims and objectives are important?’; ‘do you believe it is appropriate to use NHS data in this way?*’*; ‘do you think this is an appropriate way to handle and protect patient data?’; ‘under these circumstances, is it appropriate for trained NHS staff who would not normally have access to it (ie, not part of the routine care team) to handle data in this way?*’*; ‘is there anything else you would like to see included in the design of this study?’*. Data on prior history of TIA/stroke were also collected.

### Study oversight committee

Respondents to one-to-one interviews, focus groups and online survey were invited to join a study oversight committee. Three individuals affected by stroke and three members of the general public were recruited and met via online video conference on a sixth monthly basis. The purpose of this committee is to provide an independent review of study progress and ensure ethical handling, storage and sharing of data. Particularly, we look to explore opportunities relating to third-party collaboration and data access to bona fide researchers.

### Storage of data

Focus group and interview sessions were audio-recorded and stored as an MP3 file on an encrypted and password-protected device. Online survey data were stored on the Qualtrics XM^16^ platform under an encrypted password-protected server. Data collection, handling and storage were compliant with guidance outlined by the local NHS trust, University of Plymouth^12^ and the GDPR.^17^

^11^ ^12^

### Patient and public involvement

Patients and/or the public were not involved in the design, conduct, reporting or dissemination plans of this research.

## Results

### Focus group

A total of 16 participants attended the two focus groups. Seven attended online and nine in person. The mean age was 67. 65% were male. 36% had a history of stroke/TIA, and 57% had close friends or family affected by stroke/TIA. 7% had no experience of stroke. There were eight participants for the first focus group and nine for the second. Two withdrew participation on the day as they did not feel confident in contributing due to cognitive and communication difficulties.

1. Is it acceptable to collect, store and analyse data in this manner for medical research from: (a) within the routine clinical care team and (b) from outside the routine clinical care team?
  Within the routine clinical care team?
  All participants responded with ‘YES’. Participants felt that the research was worthwhile and that the measures taken to maintain participant confidentiality and protect personal data were sufficient.
  Outside of the routine clinical care team?
  All participants responded with ‘YES’, however, two of these specified that they felt data should not be shared with commercial entities, such as medical insurance, technology or pharmaceutical firms. Instead, they felt the data should be used for research purposes only. Participants did not object to data being shared with researchers from other regions within the UK. At this time, these participants were reassured that their data would only be used for medical research purposes and not shared with commercial organisations.
2. Has sufficient opportunity for participants to ‘opt out’ of research been given?
  All participants felt that sufficient opportunity to opt out was provided via NDOO^10^ and the study website.^7^ In order to improve community outreach, three participants suggested that opt-out could be advertised using posters in GPs, supermarkets and post offices. Two participants also suggested creating an additional ‘opt-in’ register, allowing those who had signed up to the NDOO to opt their data into the project.
3. Is there anything further you would like to add?
  Participants wished the project well, and many expressed gratitude for the research, given their personal experience with stroke. Two participants volunteered to join an oversight committee for further PPI in the project.
  Potential impacts for the research were also briefly discussed. This included discussion around designing a clinical trial in which individuals who are identified as high risk of stroke could be treated with a placebo versus a pharmaceutical and/or lifestyle intervention. All participants felt this would be appropriate and valuable research.

### One-to-one interview

16 individuals were approached in the inpatient setting. 14 agreed to participate in a one-to-one interview. 12 had a history of stroke, and two cared for someone with a stroke. 57% were male. The mean age was 71. The mean interview time was 9 min 32 s.

1. Is it acceptable to collect, store and analyse data in this manner for medical research from: (a) within the routine clinical care team and (b) from outside the routine clinical care team?
  All participants responded with ‘YES’. Participants generally felt that handling data in this way was justified by the stated medical purpose, namely, to improve stroke care. No participant objected to sharing this type of data outside of the routine clinical care team; however, one participant stated a preference for the data to remain within the UK. Another participant also specified that they would only want data relating to their health to be collected, and not other forms of personal data (eg, financial).
2. Has sufficient opportunity for participants to ‘opt out’ of research been given?
  All participants felt that sufficient opportunity to opt out was provided via NDOO^10^ and the study website.^7^ They were satisfied with the current methods of advertising the study via the local NHS trust’s social media. Three participants suggested advertising the project via the ‘Stroke Association’^13^ and ‘Age UK’.^18^
3. Is there anything further you would like to add?
  Participants generally used this opportunity to express their support for the study. One participant stressed that they felt the data should be used for academic research only, and not sold/shared with insurance, technology or drug companies.

### Online survey

**Figure 2:**
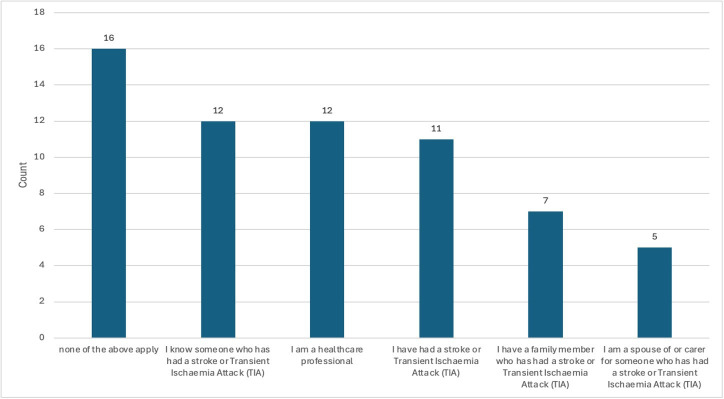
Survey respondent demographics.

There were 61 respondents to the online survey. 17% had a history of stroke/TIA, and 38% had friends or family affected by stroke (figure 2). 36% had no lived experience of stroke.

60 of 61 respondents felt the study aims and objectives were important. 60 of 61 respondents felt this was an appropriate way to use NHS data. Three participants commented that they were reassured that the data would remain within the NHS or the University. 60 of 61 felt it was appropriate for data to be handled by members within the routine clinical care team. 59 of 62 felt it was appropriate for data to be handled by members outside of the routine clinical care team. Two participants commented that they felt that the stated medical purpose justified such unsolicited data access. One participant commented that ‘as long as the fewest number of people have access’. One participant responded ‘no’ to all answers, but did not specify why. The additional comments respondents made to survey questions are summarised in table 1.

**Table 1:** Survey responses (comments)

Study aims and objectives are important?	Appropriate to use NHS data in this way?	Appropriate for data to be handled within the routine clinical care team?	Appropriate for data to be handled outside of the routine clinical care team?
I think it is very important to find AI tools to identify more and more conditions. AI can be very accurate, and from patient data, new studies can be done	I think it is a good way to analyse and use patient data	I think it is a very good way to manage patients’ opt out and consent. Historical data don't need patient consent anyway, and the data will be deidentified	Without this, progress would be hindered
It's always better to be prepared as much as possible, so identifying previously unknown risk factors for stroke is a very good plan!	Anything that can help detect risks before any TIA or stroke is both good for the patient/family and the NHS	No, it seems covered. Information governance is important, but you need the data to avoid future health issues	The purpose justifies this access
I think the objectives are a great idea, minimising the risk of stroke is important	Sounds like it will stay within the NHS, in which case this is fine		As long as the fewest number of people have access
Anything that can prevent people from having a stroke would be an amazing thing	If we can reduce stroke, it will prevent untold misery		I would be concerned about patient confidentiality
Seems like an important project	Yes. I am reassured that it stays within the NHS		
Very important	I am sure the data will be suitably protected and anonymised		
I support all medical studies that seek to prevent health conditions and aim to increase the health of the population and save resources used for recovery	Think it's a great proactive idea. Mum had a major stroke at 43, which could have been prevented. I'm keen to prevent myself from having one		
	Seems reasonable given the study aims		

AI, artificial intelligence; NHS, National Health Service; TIA, transient ischaemic attack.

### Study oversight committee

Thus far, our oversight committee has met on two occasions to discuss study progress and third-party data access. Participants felt that sharing of anonymised data with bona fide researchers and some commercial organisations was appropriate, assuming the research would be of benefit to patients and no patient identifiers would be transferred. Possible data sharing frameworks that were discussed include sharing via a secure data environment or using a federated learning model. Future meetings will aim to decide on a preferred data sharing framework and the extent of data that should be shared with external researchers.

### Amendments to study design based on PPI feedback

Following feedback obtained via our PPI work, three major opportunities for improvement were identified:

First, our strategy in publicising opting out of our project has mainly focused on the inpatient hospital setting and online social media. However, participants’ feedback identified that those ‘less computer literate’ or healthier populations who have only attended the hospital on one-off occasions may not be reached via these platforms. Hence, we have further publicised the project via posters in community spaces such as supermarkets, GP surgeries and post offices to widen our catchment. Based on participant feedback, we have also reached out to ‘Age UK’^18^ and the ‘Stroke Association’^13^ to advertise the project online and via the post.

Second, participants felt that many individuals may be unsure of their data-sharing preferences and hence suggested creating a local ‘opt-in’ register. A register was therefore created to enable patients and the public to opt their data into the project. The details on how to opt in were advertised via poster, social media and the study website.

Finally, our creation of a study oversight group has enabled us to explore the feasibility of expanding our dataset through collaboration with third parties and discuss frameworks to share anonymised data with bona fide researchers.

## Discussion

In this article, we publish the findings of the PPI work for the ABSTRACT study.^7^ We evaluated the opinions of a total of 83 volunteers on using AI to predict future risk of stroke from routine healthcare data, and in acquiring this data via an opt-out consent model. This was done using a combination of patient interviews, focus groups and online survey. To our knowledge, no other study has sought to investigate the opinions of the general public and patients on using AI to predict the future risk of stroke.

Nearly all participants felt that this was an appropriate use of NHS data and that it was acceptable for the data to be handled by members both in and outside of the routine clinical care team. Nearly all participants also felt that handling these data without gaining explicit patient consent was justified by the stated medical purpose of preventing future stroke. Likewise, they supported the use of an opt-out system.

Based on our study protocol, participants generally also felt that sufficient opportunity was given to opt out; however, several participants suggested advertising study posters in public places such as GP surgeries, supermarkets and post offices. Only one participant (online survey) felt that the study aims were not important and did not feel that this was an appropriate use of NHS data, but did not explain why.

These consensuses were based on several conditions. First, the data would be used for research purposes only and not sold to third-party organisations. Second, the data would be handled in compliance with GDPR guidance^17^ and only stored on encrypted NHS and/or university devices. And finally, the data would be collected from the minimum number of participants and handled by the minimum number of researchers.

With respect to AI in medical research, these findings are in agreement with several other studies that have demonstrated a favourable public opinion in using AI in medical research, but were sceptical about the accuracy, accountability and ethico-legal implications of AI in healthcare.^619^,^21^ The common concerns that were raised in both this and other studies included cybersecurity, data leaks and the sale of data to commercial bodies (which might impact health insurance, mortgage, etc).^620^,^22^ However, our cohort generally felt that the potential benefits of this research outweighed these risks, assuming data handling was compliant with GDPR and the data would not be sold to commercial bodies. This is consistent with a recent UK government survey that identified public concerns around AI could be mitigated with public education, project transparency and adherence to ethical, legal and governance frameworks.^6 22^

Previous research has also highlighted concerns of AI dehumanising medical care and threatening job security.^5 19^ Since the accuracy of an AI algorithm is largely dependent on the quality of the training data, concerns have also been raised about the reliability of AI-driven medical decision-making, especially in minority groups who may be under-represented in the training data set.^23^ None of our participants volunteered these concerns; however, they were not explicitly discussed.

With regard to consent, other research has evaluated the public opinion of adopting an opt-out strategy in consenting for medical research.^24^,^26^ Our findings are consistent with this research in that both the general public and patients are generally supportive of opt-out models of consent. However, again, this is often on condition that projects maintain transparency, adhere to ethical and legal frameworks (such as GDPR) and that it is perceived that the research will provide clear health benefits.^27^,^29^ The latter of which is highly influenced by public and patient education, in addition to their trust in the institution performing the research.^30^

### Limitations

Our project was subject to several limitations. First, when recruiting for a focus group, many stroke patients were unable to attend in person or virtually. Some also did not feel confident in contributing due to cognitive or communication difficulties. Consequently, those with advanced disability or without access to a computer may be under-represented. However, we attempted to mitigate this under-representation by performing one-to-one interviews with stroke patients in the hospital setting.

Second, of our 83 participants, only 19% reported no lived experience of TIA or stroke, and most were from the Cornwall and South Devon, UK area. Hence, these findings may not be generalisable to the entire UK/world population and may under-represent members of the public who have not had their views shaped by personal experiences with TIA or stroke.

Third, given that the participants generally held favourable views of our methods, little information was obtained about why individuals may object to using AI in medical research or adopting an opt-out consent model, making it difficult to understand ways to mitigate these barriers.

Finally, we assessed these views in the context of our ongoing project, ABSTRACT, which aims to use AI to predict future risk of stroke from routine hospital data.^7^ Hence, the views highlighted here may not be representative of views held for similar or other AI-based medical research projects. Assessment of project risk versus benefit and transparency is subjective and may vary between communities, highlighting the need for project-specific PPI work.

## Conclusion

We believe that this research identifies two relevant findings. First, both the general public and those with lived experience of stroke appear supportive of the use of large, deidentified medical datasets to train AI models to predict future risk of stroke. Participants also supported the acquisition of these datasets via an ‘opt-out’ consent model, but this was under the condition that the research process is transparent, adherent to ethical and legal frameworks, aims to provide clear health benefits and sufficient opportunity is given to opt out.

### Future research

We believe these findings offer an up-to-date perspective for ethical reviewers when considering medical research involving AI and opt-out models of consent. Although patient and public support appears favourable, an enhanced understanding of why people may object to AI in medical research is needed in order to further AI governance and improve public trust. We also accept that the sample of this research was not representative of the diversity of the UK; therefore, additional work is required in communities where it is known there is a higher level of distrust of medical research involving large datasets.^31^ Also, given that the majority of our respondents had been either personally affected or knew someone affected by TIA or stroke, further evaluation of opinions held by members of the public with no lived experience of TIA or stroke is required.

### Ethical approval and consent

Written and/or oral consent was obtained from all participants. Ethics and data handling procedures complied with the University of Plymouth’s ‘research ethics and integrity policy’^11^ and ‘research data management policy’.^12^ This study involves human participants and ethical approval for this study was obtained from the Health Research Authority (South Central—Oxford B Research Ethics Committee. CAG reference 25/CAG/0021: REC reference: 23/SC/0217. IRAS 306246).

## Supplementary material

10.1136/bmjopen-2025-108671 online supplemental file 1

10.1136/bmjopen-2025-108671 online supplemental file 2

## Data Availability

Data are available upon reasonable request.
