# Supplementary material for: Opinions of the UK general public and stroke survivors in using artificial intelligence and ‘opt-out’ models of consent in medical research: a qualitative study
Source: BMJ Open. 2025 Dec 15;15(12):e108671. doi: 10.1136/bmjopen-2025-108671 (PMC12706208; doi:10.1136/bmjopen-2025-108671)
Supplement: Supplementary file 3 [file bmjopen-2025-108671.reviewer_comments.pdf]

PEER REVIEW HISTORY

BMJ Open publishes all reviews undertaken for accepted manuscripts. Reviewers are asked to complete a checklist review form and are provided with free text boxes to elaborate on their assessment. These free text comments are reproduced below.

ARTICLE DETAILS

Title (Provisional)

Opinions of the UK general public and stroke survivors in using artificial intelligence and “opt-out” models of consent in medical research: a qualitative study.

Authors

Heseltine-Carp, William; Thurtson, Mark; Allen, Michael; Browning, Daniel; Courtman, Megan; Kasabe, Aishwarya; Ifeachor, Emmanuel; Mullin, Stephen

---

VERSION 1 - REVIEW

**Reviewer**	**1**
**Name**	**Rose, David**
**Affiliation**	**USF Health Morsani College of Medicine, Neurology**
**Date**	**15-Sep-2025**
**COI**	**None**

---

interesting concept however a few items need to be addressed in this paper:

1. abstract background - how do you know 20-30% of public is skeptical of AI? From what dataset did you obtain that number, please refer to it (directly in the background sentence). Also that number range seems a bit low, are there other data in the literature on this perhaps?

2. abstract aims - what is ABSTRACT UK MRC project exactly? Need a little more info on that here in the abstract.

3. abstract methods - how were opinions gathered? Dont need extensive explanation but there is no actual method in the methods section, ironically.

4. abstract results - almost all supported what aims exactly? These need to be delineated.

5. intro - carers is a word? Did you mean caregivers?

6. intro - ensures is too strong a word here ... Did you mean enables or assists or bolsters?

7. intro - you write "Increasingly, evidence of PPI input is often required by funders and ethical review boards." but you dont cite this "evidence" -- please address.

8. intro - throughout the intro, you overuse the words "important" and "importance" -- please tone this down a bit; perhaps replace these overused words with something softer.

9. aims - exactly 120,000 patients?

10. methods - question 1 is a bit awkward with the phrase "inside and outside" - you meant from within and from outside, yes?

11. methods - question 2 is a bit awkward with "has" written twice: "Has sufficient opportunity for

participants to ‘opt out’ of research has been given?"

12. methods - question 3 is a bit awkward as a yes/no: Is there anything further you

would like to add? Because what happens with a yes answer? This isn't clearly explained here.

13. Cannot start sentence with a number: 36% had a history of stroke/TIA and

57% had close friends or family affected by stroke/TIA.7% had no experience of stroke.

14. For this question, how does this make sense if "all answered yes" but 2 "objected" -- isn't that a NO for those two people? B) Outside of the routine clinical care team, all participants responded with “YES”. However two participants stressed that they would object to the data being used for commercial purposes, particularly in the context.

15. Regarding 14, what did you say in response to the participants stressed who objected to data used for commercial purposes? Did you tell them that it would not be shared with medical insurance, tech and pharmaceutical companies, only for research purposes? Or did you not reply, only collecting their answers.

16. Shouldn't the national opt-out database acronym be NOOD instead of NDOO?

17. discussion - did you discuss with patients what exactly is AI? How it works and what is machine learning? Did you review any data with them on what AI is currently doing for stroke?

18. Here are a few manuscripts on how AI has changed stroke care already. You may want to include these to let readers know that AI is already here. Granted, it will be improving and mutating in the future but it is not a hypothetical at this point in 2025.

ref 1: "The Ghost in the Machine: Artificial Intelligence in Neurocardiology Will Advance Stroke Care." Neurohospitalist. 2024 Oct 4:19418744241288887. doi: 10.1177/19418744241288887. PMID: 39544272; PMCID: PMC11559459.

ref 2: "Viz.ai Implementation of Stroke Augmented Intelligence and Communications Platform to Improve Indicators and Outcomes for a Comprehensive Stroke Center and Network." AJNR Am J Neuroradiol. 2023 Jan;44(1):47-53. doi: 10.3174/ajnr.A7716. Epub 2022 Dec 8. PMID: 36574318; PMCID: PMC9835916.

19. Fig. 3 is pointless with 4 of the same approximate ratios.

20. Fig. 1 data flow is awkward with the thin arrows, please clarify and enlarge text regarding ongoing linkage.

**Reviewer**	**2**
**Name**	**Rubiera, Marta**
**Affiliation**	**Vall d'Hebron University Hospital, Stroke Unit**
**Date**	**13-Oct-2025**
**COI**	**None**

I read with great interest the manuscript from Heseltine-Carp et al about the opinions on AI and opt-out consent model for stroke research. It is a well written very interesting work of an issue of great relevance today. The authors have done a multimodal approach, and gathered information in different ways, which has increased the value of their results.

I only have a concern about they affirmation that they have data from general population. Most of the participants from the different sources were stroke survivors and families/friends, who are probably biased to an increased awareness of the disease. Even those without a direct contact with stroke were collected through webpages or social media of the hospitals, NHS or the project itself, and probably again are biased toward a high stroke or healthcare awareness, and high technology literacy. This needs to be clearly stated as a limitation of the study, and as commented by the authors, should lead to a new study oriented to a "more general" population, to have a wider opinion of the acceptance and the barriers for adoption of this kind of research.

VERSION 1 - AUTHOR RESPONSE

Reviewer 1

"1. abstract background - how do you know 20-30% of public is skeptical of AI? From what dataset did you obtain that number, please refer to it (directly in the background sentence). Also that number range seems a bit low, are there other data in the literature on this perhaps?"

The abstract has been updated to provide context for this figure. This was based on an ongoing UK government tracker survey, which aims to evaluate public attitudes towards data and AI. Based on wave 3 of the survey, overall approximately 20-40% were sceptical of AI having a positive societal impact (see figures 12-14). Public trust appeared much higher for academic institutions and the NHS, compared to non-academic and commercial organisations (Table 2), which may explain some of the discrepancies seen in studies evaluating public opinions of AI. These findings are consistent with a recent systematic

review, which identified that patients and the public were generally accepting of AI within healthcare, assuming there remained a degree of human supervision.

1) ‘Public attitudes to data and AI: Tracker survey (Wave 3)’, GOV.UK. Accessed: Jun. 19, 2024. [Online]. Available: https://www.gov.uk/government/publications/public-attitudes-to-data-and-ai-tracker-survey-wave-3/public-attitudes-to-data-and-ai-tracker-survey-wave-3

2) A. T. Young, D. Amara, A. Bhattacharya, and M. L. Wei, ‘Patient and general public attitudes towards clinical artificial intelligence: a mixed methods systematic review’, *The Lancet Digital Health*, vol. 3, no. 9, pp. e599–e611, Sep. 2021, doi: 10.1016/S2589-7500(21)00132-1.

“However, a recent UK government survey revealed that 20-40% of the public remain sceptical of its use in health research due to concerns about data security, patient-practitioner communication, and commercialisation of data.”

“2. abstract aims - what is ABSTRACT UK MRC project exactly? Need a little more info on that here in the abstract.”

A paragraph outlining a brief overview of the ABSTRACT project has now been added:

“The ABSTRACT[7] study aims to train ML models on routine computerised tomography (CT) head, magnetic resonance imaging (MRI) head, electrocardiogram (ECG), echocardiogram (echo), laboratory test and past medical history data, to predict future stroke risk at 1,3,5 and 10 years. Models will be trained using general practice (GP) and hospital records from 9155 stroke cases and 109,581 controls obtained from NHS sites across Devon and Cornwall in the United Kingdom (UK).”

“3. abstract methods - how were opinions gathered? Dont need extensive explanation but there is no actual method in the methods section, ironically.”

and

“4. abstract results - almost all supported what aims exactly? These need to be delineated.”

The abstract *aims* and *methods* sections have been rewritten to provide a more comprehensive overview of the project:

“Aims
We aimed to evaluate the opinions of stroke/TIA patients, caregivers and members of the public on the following themes: (1) the acceptability of using AI to predict stroke from electronic health records, (2) obtaining these data using an opt-out model of consent, and (3) allowing access to this dataset from members both within and outside of the routine clinical care team.

Methods
A total of 83 participants were recruited via NHS social media and approaching hospital inpatients. Participants were first provided with background information on stroke, AI in

medical research and ABSTRACTs proposed data handling protocol. A mixed methods approach was then used to explore each of the above themes using online survey, semi-structured focus groups, and one-to-one interviews."

"5. intro - carers is a word? Did you mean caregivers?"

This has been updated to "caregivers":

"Patient and public involvement (PPI) describes the process of actively engaging patients, caregivers and the general public in designing, conducting and disseminating research."

"6. intro - ensures is too strong a word here ... Did you mean enables or assists or bolsters?"

This has been replaced with the term "encourages":

"Secondly, it encourages research to be conducted in an ethical and transparent manner."

7. intro - you write "Increasingly, evidence of PPI input is often required by funders and ethical review boards." but you dont cite this "evidence" -- please address.

The following reference has now been added to this statement.

Absolom K, Holch P, Woroncow B, Wright EP, Velikova G. Beyond lip service and box ticking: how effective patient engagement is integral to the development and delivery of patient-reported outcomes. Qual Life Res. 2015 May;24(5):1077-85. doi: 10.1007/s11136-014-0909-z. Epub 2015 Jan 11. PMID: 25577498.

"8. intro - throughout the intro, you overuse the words "important" and "importance" -- please tone this down a bit; perhaps replace these overused words with something softer."

As suggested, these sentences have been reworded with softer wording:

"This further highlights the need for researchers to undertake such work in order to produce ethical and high-quality research[2]."

"High-quality PPI work is therefore an increasingly necessary prerequisite to this type of research."

"9. aims - exactly 120,000 patients?"

This has been updated to the true number of participants in the ABSTRACT study:

"Models will be trained using general practice (GP) and hospital records from 9155 stroke cases and 109,581 controls obtained from NHS sites across Devon and Cornwall in the United Kingdom (UK)."

"10. methods - question 1 is a bit awkward with the phrase "inside and outside" - you meant from within and from outside, yes?"

Throughout the manuscript, the phrasing of question 1 has been corrected to:

"Is it acceptable to collect, store and analyse data in this manner for medical research from: a) within the routine clinical care team and b) from outside the routine clinical care team?"

"11. methods - question 2 is a bit awkward with "has" written twice:"

Throughout the manuscript, the phrasing of question 2 has been corrected to:

"Has sufficient opportunity for participants to 'opt out' of research been given?"

12. methods - question 3 is a bit awkward as a yes/no: Is there anything further you would like to add? Because what happens with a yes answer? This isn't clearly explained here.

The description of the focus group methods has been updated to clarify how responses to each of the questions are handled:

"For questions one and two, a consensus was reached and summarised as "(YES/NO/UNABLE TO REACH CONSENSUS)". Those who responded "yes" to question three were given the opportunity to freely record additional comments or discussion points on the project."

"13. Cannot start sentence with a number: 36% had a history of stroke/TIA and 57% had close friends or family affected by stroke/TIA.7% had no experience of stroke."

Throughout the manuscript, the styling has been updated to express numbers in words over digits at the start of a sentence. For example:

"Thirty-six percent had a history of stroke/TIA and 57% had close friends or family affected by stroke/TIA."

"14. For this question, how does this make sense if "all answered yes" but 2 "objected" -- isn't that a NO for those two people? B) Outside of the routine clinical care team, all participants responded with "YES". However two participants stressed that they would object to the data being used for commercial purposes, particularly in the context."

And

"15. Regarding 14, what did you say in response to the participants stressed who objected to data used for commercial purposes? Did you tell them that it would not

be shared with medical insurance, tech and pharmaceutical companies, only for research purposes? Or did you not reply, only collecting their answers."

All participants responded with "yes", however, two of these participants specified that they would not want their data shared with commercial entities, such as medical insurance, technology or pharmaceutical firms. At this time, we reassured these two participants that their data would be used for medical research purposes only and not shared with commercial organisations.
The results section of the focus group has been updated to more clearly reflect this finding:

A) "Outside of the routine clinical care team?
All participants responded with "YES", however, two of these specified that they felt data should not be shared with commercial entities, such as medical insurance, technology or pharmaceutical firms. Instead, they felt the data should be used for research purposes only. Participants did not object to data being shared with researchers from other regions within the UK. At this time, these participants were reassured that their data will only be used for medical research purposes and not shared with commercial organisations."

"16. Shouldn't the national opt-out database acronym be NOOD instead of NDOO?"

The manuscript has been updated to correctly define the "national data opt-out" service (NDOO).

"Participants were then compared against the national data opt-out database (NDOO)[10] and removed accordingly."

"17. discussion - did you discuss with patients what exactly is AI? How it works and what is machine learning? Did you review any data with them on what AI is currently doing for stroke?"

In the days prior to obtaining opinions, participants were provided with information packs on the definition of stroke, stroke risk factors, the definition of AI and its roles in stroke research, the aims of the ABSTRACT project and ABSTRACTs approach in acquiring and handling data. This documentation can be found in appendices 1 and 2.
On the day of the one-to-one interviews and focus groups, the above topics were revisited with participants, where they were also given the opportunity to clarify or explore any of these topics in more detail.

A paragraph has been added to the start of the methods section to outline the above:

"Prior to any of these three approaches, participants were provided with two documents. The first provided background information on the definition of stroke and stroke risk factors, the definition of AI and its roles in stroke research, the aims of the ABSTRACT project, and finally, the purpose of PPI in research (appendix 1). The second provided details on how our study planned to acquire, handle, process and store clinical data (appendix 2)."

"18. Here are a few manuscripts on how AI has changed stroke care already. You may want to include these to let readers know that AI is already here. Granted, it will be improving and mutating in the future but it is not a hypothetical at this point in 2025. ref 1: "The Ghost in the Machine: Artificial Intelligence in Neurocardiology Will Advance Stroke Care." Neurohospitalist. 2024 Oct 4:19418744241288887. doi: 10.1177/19418744241288887. PMID: 39544272; PMCID: PMC11559459. ref 2: "Viz.ai Implementation of Stroke Augmented Intelligence and Communications Platform to Improve Indicators and Outcomes for a Comprehensive Stroke Center and Network." AJNR Am J Neuroradiol. 2023 Jan;44(1):47-53. doi: 10.3174/ajnr.A7716. Epub 2022 Dec 8. PMID: 36574318; PMCID: PMC9835916."

Thank you for these references; these have now been added to support the statement on *"It is likely that AI will continue to accelerate medical research and transform clinical practice[3][4]."*

"19. Fig. 3 is pointless with 4 of the same approximate ratios."

Figure 3 has now been removed from the manuscript.

"20. Fig. 1 data flow is awkward with the thin arrows, please clarify and enlarge text regarding ongoing linkage."

Figure 1 has been updated with a simpler and clearer illustration of data flow and acquisition for the ABSTRACT project.

Reviewer 2

"I only have a concern about they affirmation that they have data from general population. Most of the participants from the different sources were stroke survivors and families/friends, who are probably biased to an increased awareness of the disease. Even those without a direct contact with stroke were collected through webpages or social media of the hospitals, NHS or the project itself, and probably again are biased toward a high stroke or healthcare awareness, and high technology literacy. This needs to be clearly stated as a limitation of the study, and as commented by the authors, should lead to a new study oriented to a "more general" population, to have a wider opinion of the acceptance and the barriers for adoption of this kind of research."

This is an important point that has now been highlighted in the limitations section. Furthermore, a statement outlining the need for future research to assess the opinions of those with no lived experience of TIA or stroke in using health data to build AI stroke prediction models has been added to the "future research" section.

"Secondly, of our 83 participants, only 19% reported no lived experience of TIA or stroke, and most were from the Cornwall and South Devon, UK area. Hence, these findings may not be generalisable to the entire UK/world population and may

underrepresent members of the public who have not had their views shaped by personal experiences with TIA or stroke. "

"Also, given that the majority of our respondents had been either personally affected or knew someone affected by TIA or stroke, further evaluation of opinions held by members of the public with no lived experience of TIA or stroke is required. "

VERSION 2 - REVIEW

**Reviewer**	**1**
**Name**	**Rose, David**
**Affiliation**	**USF Health Morsani College of Medicine, Neurology**
**Date**	**04-Nov-2025**
**COI**	

---

revisions complete on my side
